# Angiotensinase C mRNA and Protein Downregulations Are Involved in Ethanol-Deteriorated Left Ventricular Systolic Dysfunction in Spontaneously Hypertensive Rats

**DOI:** 10.1155/2015/409350

**Published:** 2015-10-05

**Authors:** Jinyao Liu, Ayako Hakucho, Tatsuya Fujimiya

**Affiliations:** Department of Legal Medicine, Yamaguchi University Graduate School of Medicine, Ube, Yamaguchi 755-8505, Japan

## Abstract

The influences of angiotensinase C on ethanol-induced left ventricular (LV) systolic function were assessed in spontaneously hypertensive rats (SHRs). SHRs were fed by a liquid diet with or without ethanol for 49 days. The normotensive Wistar Kyoto rats (WKY) were fed by the liquid diet without ethanol and used as control. We evaluated LV systolic function, angiotensinase C mRNA and protein expressions, activation of the renin-angiotensin system (RAS), and the gene expressions of LV collagen (Col) III a1 and matrix metalloproteinases- (MMP-) 9. Compared to the WKY, LV systolic dysfunction (expressed by decreased fractional shortening and ejection fraction) was observed in the SHRs before ethanol treatment and further deteriorated by ethanol treatment. In the ethanol-treated SHRs, the following were observed: downregulations of angiotensinase C mRNA and protein, increased RAS activity with low collagen production as evidenced by angiotensin II and angiotensin type 1 receptor (AT_1_R) protein upregulation, AT_1a_R mRNA downregulation, and an MMP-9 mRNA expression upregulation trend with the downregulation of Col III a1 mRNA expression in LV. We conclude that chronic ethanol regimen is sufficient to promote the enhanced RAS activity-induced decrease in the production of cardiac collagen via downregulated angiotensinase C, leading to the further deterioration of LV systolic dysfunction in SHRs.

## 1. Introduction

Chronic heavy alcohol consumption is a common cause of heart failure and it leads to approximately one-fifth of all sudden cardiac deaths [1]. The underlying mechanisms through which alcohol produces this condition remain poorly understood [2].

Hypertensive heart disease, the leading cause of death from hypertension, causes left ventricular hypertrophy (LVH) through neural and humoral factors [3, 4]. As does compensatory cardiomyocyte hypertrophy, myocardial fibrosis makes a considerable contribution to LVH and leads to the development of LV diastolic and systolic dysfunction and ultimately to heart failure [5]. The activation of renin-angiotensin system (RAS) is a significant risk factor for the development of arterial hypertension, LVH, and heart failure [6–8]. Components of the RAS have been detected at both the cardiac mRNA and protein levels [9], and angiotensin II, the final mediator of the RAS, has been implicated in the production of matrix metalloproteinases (MMPs) and the breakdown of collagen [10]. In the spontaneously hypertensive rat (SHR), a widely studied animal model of human essential hypertension, MMPs damage cells directly by inducing the cleavage of the extracellular domain of several key receptors, resulting in the diverse cell dysfunctions characteristic of SHR [11]. Enhanced RAS activity thus acts on several different components of extracellular matrix formation and deposition to influence the matrix turnover that is responsible for the production of collagen and finally leads to cardiac dysfunction. However, the role of RAS in the development of alcohol-induced LV systolic function in essential hypertensive heart requires further investigation.

Angiotensinase C, also known as prolylcarboxypeptidase (PRCP) and reported to have antihypertensive and antiproliferative roles via inactivation of the RAS, is responsible for RAS activity by the degradation of angiotensin II, the final mediator of the RAS [12]. The functions of angiotensinase C include the hydrolysis of angiotensin II to angiotensin 1–7 [13], which play a vital role in cardiac hypertrophy and remodeling [14–16].

SHR is hypertensive rat and that itself contributes to the cardiac remodeling and hypertrophy with the reduced cardiac angiotensinase C gene and protein expressions [17]. However, it is not known if the gene defect itself leads to specific heart defects in alcoholics. The present study thus provides, for the first time, direct evidence that enhanced RAS activity may be involved in the chronic ethanol consumption-induced development of LV systolic dysfunction via an angiotensinase C-dependent pathway in the essential hypertensive heart.

## 2. Methods

### 2.1. Animal and the Chronic Ethanol Treatment

Seven-week-old male normotensive Wistar Kyoto rats (WKY) (*n* = 6) and 7-week-old male SHRs (*n* = 13) were purchased from Japan SLC (Hamamatsu, Shizuoka, Japan). The rats were housed in a temperature-controlled room on a 12 hr light/dark cycle at the Institute of Laboratory Animals of Yamaguchi University. All rats were fed a nutritionally adequate liquid diet originally formulated by Lieber and DeCarli, purchased from Oriental Yeast Co., (Tokyo). The rats were divided into control liquid diet-fed WKY (WKY, *n* = 6), control liquid diet-fed SHR (SHR, *n* = 6), and ethanol liquid diet-fed SHR (SHR + Et, *n* = 7) groups. The protocol used for chronic ethanol exposure was as described [18]. Briefly, at the beginning of the study, all rats were given the control liquid diet and standard rat pellet chow for 4 days. For the next 4 days, the rats were fed either the control liquid diet or the ethanol liquid diet at a concentration of 3 g/dL. The control liquid diet-fed rats (WKY and SHR groups) received the further control liquid diet for 45 days, whereas the ethanol group (SHR + Et) received a diet containing 4 g/dL ethanol for the next 3 days, followed by 5 g/dL for 42 days, resulting in an overall exposure to ethanol of 49 days.

All animal experiments were performed according to the protocol approved by the Ethics Committee on Animal Experiments of the Yamaguchi University School of Medicine and were controlled by the committee's guidelines for animal experiments (#13-012).

### 2.2. Measurements of Arterial Blood Pressure (BP) and LV Functional Performance

Before and at the end of the experiments, the arterial blood pressure (BP) was measured in conscious rats by a tail-cuff monitoring system (BP-98A, Softron Co., Tokyo), and the LV systolic functional performance was evaluated by echocardiography (echocardiograph model SSD-1000 with a 10 MHz sector scan probe, Hitachi Aloka Medical, Tokyo) in anesthetized rats as described [18]. To avoid the influence of acute ethanol consumption, BP measurements and echocardiographic studies were performed between 2:00 and 4:00 p.m. in the ethanol group as described [19].

Systolic, diastolic, and mean BP values were recorded; however, only the mean blood pressure (MBP) data were used for the statistical analysis.

The internal LV end-diastolic diameter (LVDd) and the LV end-systolic diameter (LVDs) were measured according to the recommendations of the American Society of Echocardiography [20]. LV fractional shortening (LVFS) was calculated using the following formula: LVFS = (LVDd − LVDs)/LVDd × 100%. The LV end-diastolic and end-systolic volumes were calculated using the Teichholz formula, and the LV ejection fractions (LVEFs) were obtained.

### 2.3. Preparation of Tissue Samples

After the BP measurements and echocardiographic study, the rats were weighed and anesthetized with 1%-2% isoflurane in oxygen as described [18]. The heart was removed intact and the LV was then dissected and weighed. The LV weight was calculated by dividing the LV by the body weight of each animal. Each LV was excised and was cut perpendicular to the apex-to-base axis into three pieces. Two of these pieces were immediately frozen in liquid nitrogen and stored at −80°C for later mRNA and protein analyses.

### 2.4. Real-Time Reverse Transcriptase Polymerase Chain Reaction (RT-PCR)

The total RNA was extracted from the LV tissue using the RNeasy Fibrous Tissue Mini Kit (Qiagen, Tokyo). ReverTra Ace qPCR RT Master Mix with gDNA Remover was used to remove gDNA and to synthesize cDNA according to the manufacturer's instruction. Real-time reverse transcriptase polymerase chain reaction (RT-PCR) was performed for the quantitative assessment of mRNA expression using an Applied Biosystems StepOne system (Applied Biosystems, Foster City, CA, USA). The gene expression assays for angiotensinase C (ID: Rn01511011_m1), angiotensin type 1a receptor (AT_1a_R, ID: Rn01435427_m1), angiotensin type 2 receptor (AT_2_R, ID: Mm01341373_m1), MAS-related G protein-coupled receptor D (MrgD, ID: Rn01785783_s1), matrix metalloproteinases-9 (MMP-9, ID: RnRn01423075_g1), collagen III a1 (Col III a1, ID: Rn01437681_m1), and GAPDH (ID: Rn99999916_s1) were purchased from Applied Biosystems. The relative expression genes were normalized to the amount of the GAPDH mRNA in an identical cDNA sample, using the comparative quantitative method recommended by the manufacturer; the relative expression is expressed as the fold change from the values of WKY.

### 2.5. Western Blot Assay

LV tissues harvested at the end of the study were homogenized using the Protein Extract Transmembrane Protein Extraction Kit (Novagen, Merck, Darmstadt, Germany) to extract the myocardial cytoplasm and transmembrane fraction proteins, respectively, according to the manufacturer's recommendations. Cytoplasm fraction proteins were used to evaluate the cardiac renin, angiotensin II, and angiotensinase C protein expressions. Transmembrane fraction proteins were used to evaluate the angiotensin type 1 receptor (AT_1_R), angiotensin type 2 receptor (AT_2_R), and G protein-coupled receptor-1 Mas subfamily (MAS1L) protein expressions. The protein concentration was measured by the Bradford method.

Proteins of the LV were separated by sodium dodecyl sulfate polyacrylamide gel electrophoresis (SDS-PAGE, 10% (w/v) gel for cytoplasm fraction proteins and 4–20% (w/v) gel for transmembrane fraction proteins) and transferred to a polyvinyl difluoride membrane. Each blot was incubated with anti-GAPDH (1 : 200, rabbit polyclonal; Santa Cruz Biotechnology, Santa Cruz, CA), anti-renin (1 : 500, rabbit polyclonal; Abcam, Cambridge, MA), anti-angiotensin II (1 : 200, rabbit polyclonal; Bioss, Woburn, MA), anti-AT_1_R (1 : 100, rabbit polyclonal; Santa Cruz Biotechnology), anti-AT_2_R (1 : 100, rabbit polyclonal; Santa Cruz Biotechnology), anti-MAS1L (1 : 200, rabbit monoclonal; Abcam, Cambridge, MA), and anti-angiotensinase C (1 : 200, rabbit polyclonal; Santa Cruz Biotechnology) and then incubated with the appropriate secondary horseradish peroxidase-conjugated anti-rabbit IgG antibodies. Finally, the Western blotting bands were analyzed using Quantity One software (Bio-Rad Laboratories, Hercules, CA). The reaction products of LV renin, angiotensin II, AT_1_R, AT_2_R, MAS1L, and angiotensinase C were normalized to GAPDH and are expressed as the fold change from the values of WKY.

### 2.6. Statistical Analysis

Data were collected from repeated experiments and are presented as mean ± SD. Extreme values were excluded by the Smirnov-Grubbs test. For the statistical analysis, a one-way analysis of variance (ANOVA) was used with an overall *F*-test analysis. When an *F*-value was determined to be significant by the ANOVA, the Bonferroni/Dunn post hoc test was performed for multiple comparisons. The statistical analyses were performed using Statcel2 for Windows software (OMS Publishing, Saitama, Japan). Values of *P* < 0.05 were considered significant.

## 3. Results

### 3.1. Animal Characteristics

The SHR + Et group consumed 140 ± 10 g ethanol per animal during the 49-day feeding period.

**Figure 1:**
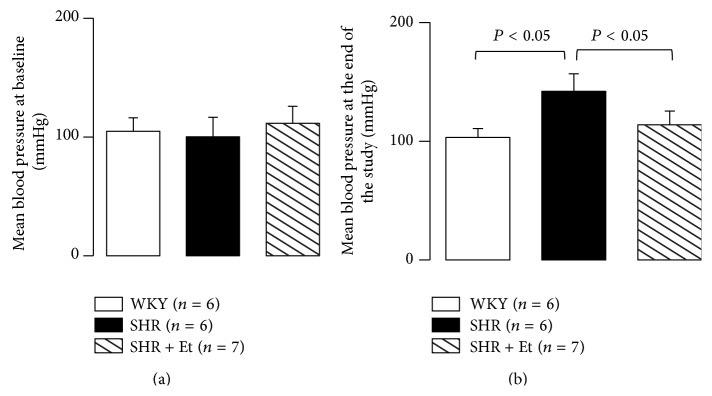
Mean blood pressure (MBP) at baseline and the end of the experiment. (a) MBP at baseline. (b) MBP at the end of the experiment. All values are means ± SDs.

No significant differences were found among the groups in MBP at baseline (7-week-old, Figure 1(a)). At the end of the feeding period (15-week-old), the SHR group showed increased MBP values with age compared to the WKY group, but the SHR + Et group showed decreased MBP values after 49-day ethanol treatment compared to the SHR group (Figure 1(b)).

**Figure 2:**
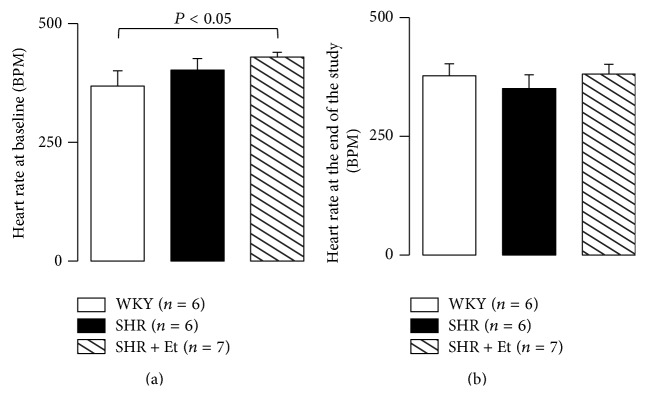
Heart rate at baseline and the end of the experiment. (a) Heart rate at baseline. (b) Heart rate at the end of the experiment. All values are means ± SDs.

Compared to the WKY group, the heart rate showed an upward trend in the SHR rats at baseline. There was a nonsignificant change in heart rate among the groups at the end of the experiments (Figures 2(a) and 2(b)).

**Figure 3:**
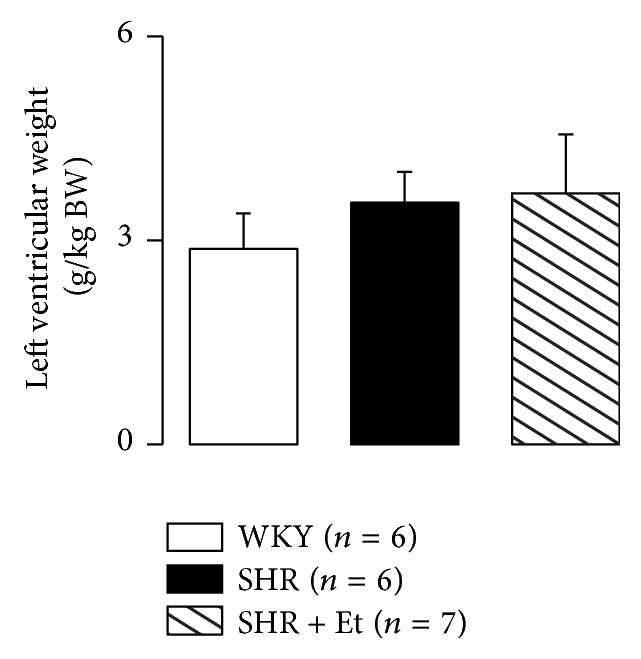
LV weight at the end of the study. All values are means ± SDs.

There was a nonsignificant change in LV weight among the groups, although there were the upward trends in both of SHR and SHR + Et groups (Figure 3).

### 3.2. Ethanol Treatment Contributed to LV Systolic Dysfunction in the SHRs

**Figure 4:**
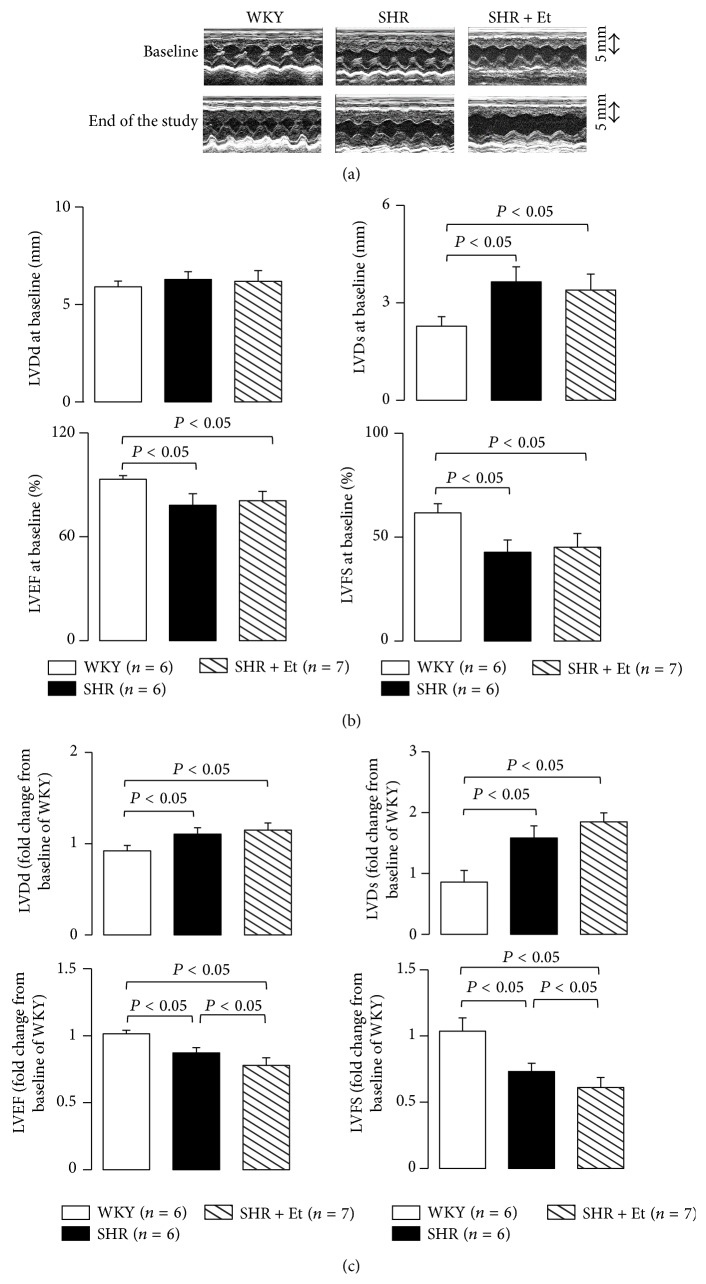
Chronic ethanol consumption damaged the LV systolic function in the SHRs. (a) Representative echocardiograms. (b) Before ethanol treatment, the SHRs showed enlarged LVDs with decreased LVFS and LVEF. (c) The LV chamber sizes were enlarged with the decreased LVFS and LVEF at the end of the experiments, especially in the SHR with 49-day ethanol treatment. All values are means ± SDs.

Representative echocardiograms and data are shown in Figure 4. Before ethanol treatment, the 7-week-old SHR (both the SHR and SHR + Et groups) showed enlarged LVDs with decreased LVFS and LVEF compared to the WKY group (Figures 4(a) and 4(b)). The LV chamber sizes (both LVDd and LVDs) were enlarged with the decreased LVFS and LVEF at the end of the experiments in the 15-week-old SHR, especially in the SHR that had undergone the 49-day ethanol treatment (Figures 4(a) and 4(c)).

### 3.3. Ethanol Was Responsible for the Lower Collagen Production in the LV Myocardium

**Figure 5:**
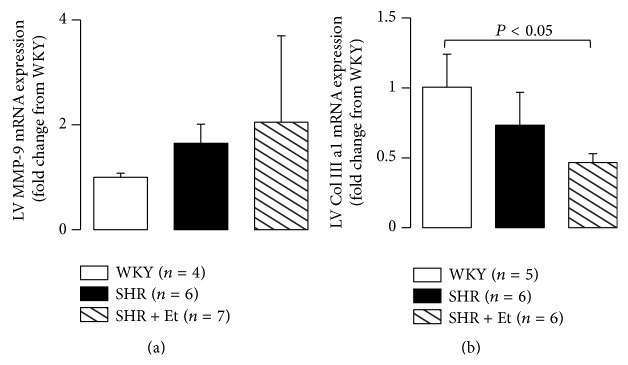
Collagen production in LV myocardium. (a) There was a nonsignificant change in MMP-9 mRNA expression among the groups, although there were the upward trends in both of SHR and SHR + Et groups. (b) Downregulated Col III a1 was observed in the ethanol-treated SHRs. All values are means ± SDs.

To determine whether the cardiac collagen production was related to the LV systolic dysfunction in the SHRs, especially those treated with ethanol, we evaluated the collagen production in LV myocardium by assessing the Col III a1 and MMP-9 mRNA expressions. There was a nonsignificant change in MMP-9 mRNA expression among the groups, although there were the upward trends in both of the SHR and SHR + Et groups (Figure 5(a)). A significant downregulation of Col III a1 (Figure 5(b)) mRNA expressions was observed in the LV myocardium of the ethanol-treated SHRs compared to the age-matched WKY rats.

### 3.4. Chronic Ethanol Consumption Enhanced the Activation of the RAS

**Figure 6:**
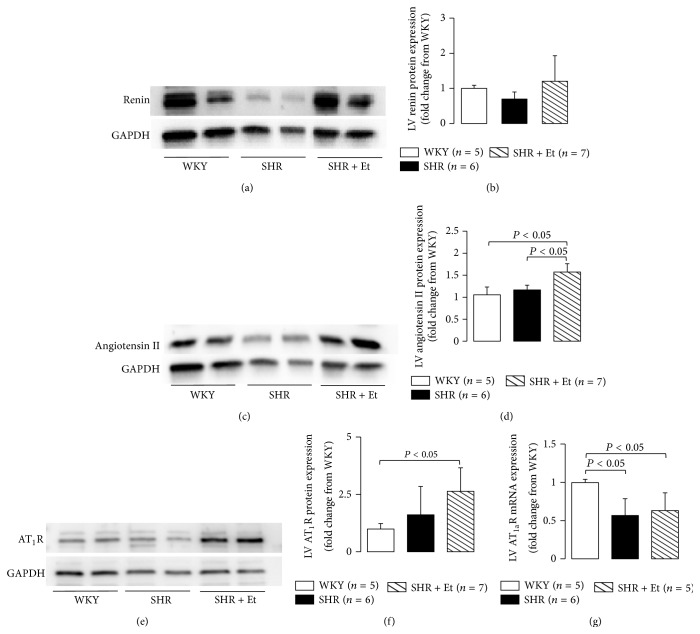
Ethanol consumption and the activation of RAS in the SHRs. (a) Representative Western blot of renin protein expression. (b) There was a nonsignificant change in renin protein expression among the groups, although there was an upward trend in the ethanol-treated SHRs. (c) Representative Western blot of angiotensin II protein expression. (d) Significant upregulated angiotensin II protein expression was observed in the ethanol-treated SHRs. (e) Representative Western blot of AT_1_R protein expression. (f) A significant upregulated AT_1_R protein expression was observed in the ethanol-treated SHRs. (g) Significant downregulated AT_1a_R mRNA expressions were observed in both of SHR and SHR + Et groups. All values are means ± SDs.

Previous experiments have shown that activation of the RAS is an important growth factor, causing cell proliferation, cell differentiation, and apoptosis [21] and that angiotensin II, the final mediator of the RAS, stimulates the production of MMPs [22]. In the present study, we measured the LV protein expressions of renin, angiotensin II, and AT_1_R and AT_1a_R mRNA expressions to evaluate the LV myocardial RAS activation. We observed an upregulating trend of renin, the significant upregulated angiotensin II, and AT_1_R protein expressions in the SHR with 49-day ethanol treatment (Figures 6(a)–6(f)). The significant downregulation of AT_1a_R mRNA (Figure 6(g)) expressions was shown in both of the SHR and SHR + Et groups. AT_1_R serves as a control point for regulating the ultimate effects of the RAS on its target tissue [23]. Our present findings indicate that ethanol was responsible for the enhanced RAS activity, which downregulated its own receptor (AT_1a_R) gene expression due to the negative feedback control, as reported [23]. We also evaluate the changes of LV gene and protein expressions in AT_2_R and receptor Mas. However, there was a nonsignificant change in mRNA expressions of AT_2_R and MrgD and protein expressions of AT_2_R and MAS1L among the groups (data not shown).

### 3.5. Downregulations of LV Angiotensinase C mRNA and Protein in SHRs

**Figure 7:**
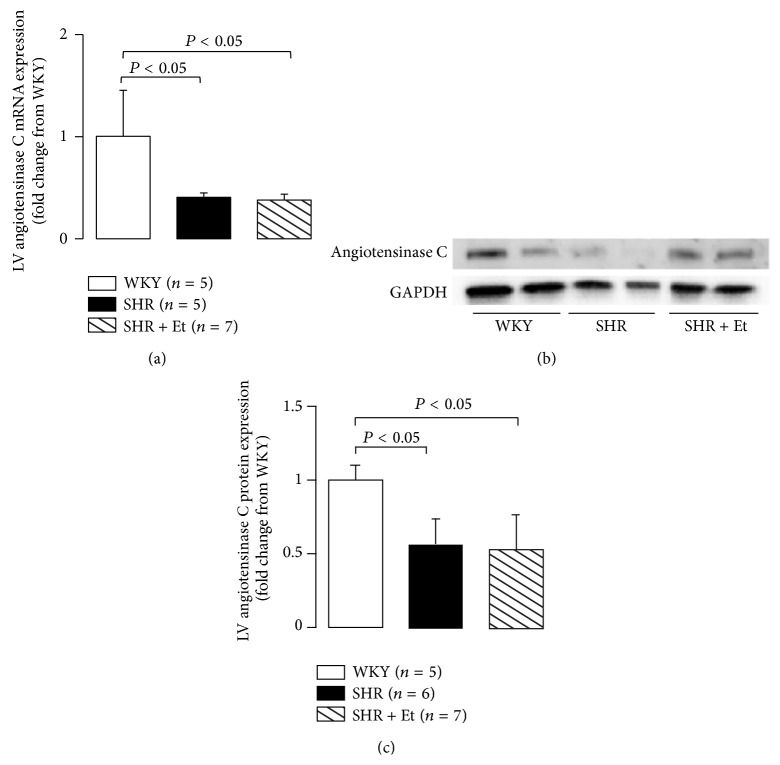
Downregulations of angiotensinase C gene and protein in LV myocardium. (a) Downregulation of angiotensinase C mRNA expressions in the SHRs. (b) Representative Western blot. (c) Downregulated angiotensinase C protein expressions in the LV myocardium of the SHRs. All values are means ± SDs.

Angiotensinase C gene mutations with loss of function induced the inadequate degradation of angiotensin II followed by blood pressure elevation with the elevated RAS activation [24]. Angiotensinase C mRNA and protein expressions were used to evaluate the LV myocardial angiotensinase C function in the present study. The angiotensinase C mRNA (Figure 7(a)) and protein (Figures 7(b) and 7(c)) expressions were significantly downregulated in the LV myocardium of the SHRs (both of SHR and SHR + Et groups) compared to the age-matched WKY rats.

## 4. Discussion

We observed that in spontaneously hypertensive rats (SHRs), 49-day 5 g/dL ethanol consumption downregulated the expressions of angiotensinase C mRNA and protein and enhanced the activation of the RAS, as evidenced by the upregulated angiotensin II and AT_1_R protein expressions, and the downregulated AT_1a_R mRNA expression, followed by LV dilation and dysfunction. This was associated with a low collagen production by the LV myocardium, expressed as a significant downregulation of Col III a1 mRNA expression. Thus, the results of the present study demonstrate the key role of angiotensinase C acting through an activated RAS-induced low collagen production in the development of LV dilation and systolic dysfunction in essential hypertensive heart with chronic ethanol consumption.

The main effect of alcohol ingestion is a loss of cardiac contractility that induces the development of dilated cardiomyopathy and leads to heart failure [2]. However, the experimental evaluation of cardiac contractile performance as alcoholism progresses has been difficult in the past due to the lack of experimental heart failure animal models that closely resemble the human condition [2]. Acute alcohol consumption is reported to have a direct cardiac depressant effect [25]. However, chronic alcohol intake has been variably reported to impair LV function [26, 27].

Myocyte orientation and myocardial fiber angles are organized and moved from the endocardium to the epicardium. It is the structural network of matrix proteins such as type I and type III collagen that provides structural integrity to adjoining myocytes and contribute to overall LV pump function through the coordination of myocyte shortening [28]. The loss of collagen fibrils and struts, which are regulated in part by the activity of MMP [29], leads to LV dilation and progressive contractile dysfunction [30]. The present study showed that chronic ethanol consumption promoted the deterioration of the impairment of LV systolic function in SHRs, and the LV systolic dysfunction was associated with a downward trend of LV collagen production, expressed as the upregulating trend of MMP-9 expression and a significant downregulation of Col III 3a1 mRNA expression.

Increased circulating levels of angiotensin II and elevated plasma renin activity occur with the development of severe heart failure in patients [31]. Chronic alcohol-induced LV dysfunction and cardiac failure have been prevented in humans by the administration of an angiotensin receptor blocker during the ingestion of alcohol, which indicates that the chronic administration of a specific AT_1_R antagonist can provide a protective effect on the LV and myocyte contractile performance during ethanol consumption by humans [31]. The effects of the enhanced RAS activity and the elevated angiotensin II on regulating the physiological processes of the cardiovascular system depend on the cellular expression and activation of AT_1_Rs. AT_1_Rs are seven-membrane superfamily of G protein-coupled receptors. The human AT_1_R gene has been mapped to chromosome. In rats, two isoforms have been pointed out: AT_1a_R on chromosome 17 and the AT_1b_R on chromosome 2; however, in vivo experiments showed that the AT_1a_R may be more important than AT_1b_R as reviewed by Mehta and Griendling [16]. Receptor Mas, a receptor for angiotensin (1–7), is an important player of the RAS. Mas-related genes are a large family of G protein-coupled receptors, which localized in chromosome 1 in the rat and chromosome 11 in humans. Angiotensin (1–7) has proved to be a weak agonist of the MAS-related G protein-coupled receptor D [32]. In the present study, chronic ethanol induced the upregulation of cardiac angiotensin II protein accompanied with the upregulated cardiac AT_1_R protein expression. However, we could not find the differences among the groups in neither cardiac AT_2_R and MrgD genes nor AT_2_R and MAS1L proteins expressions (data not shown). Contrary to heart failure due to other causes, changes in the RAS occur early in the course of chronic alcohol consumption. Consistent with past reports [33], the chronic ethanol ingestion in the present study was accompanied by a similar profile of RAS activation. An accumulation of extracellular matrix and its reduced turnover is responsible for the development of hypertrophy and heart failure [16]. Activation of the RAS is implicated in the synthesis of the extracellular matrix protein collagen via both AT_1_Rs and AT_2_Rs [34].

The production of MMPs and the breakdown of collagen are also modulated by RAS activation [10, 22]. Thus, activation of the RAS acts on several different components of extracellular matrix formation and deposition to influence matrix turnover. MMPs are zinc-dependent endopeptidases that cleave extracellular matrix proteins and affect the outcome of various physiological and pathological processes including myocardial infarction, atherosclerosis, and cardiac dysfunction. In addition to structural extracellular matrix components, MMP substrates include a multitude of ligand and receptor substrates such as cytokines, chemokines, growth factors, and adhesion molecules that alter cellular migration, adhesion, and activation [35, 36]. In SHRs, elevated MMPs cause direct damage to cells by cleavage of the extracellular domain of several key receptors, which results in the diverse cell dysfunctions characteristic of the SHR as discussed by Berry et al. in 2013 [37]. Our present data suggest that the regulation of the collagenase III a1/MMP-9 system via activation of the LV myocardial RAS is altered in the hypertrophied LV of SHRs, especially in SHRs with chronic ethanol treatment-induced systolic dysfunction.

Gene manipulation studies in animals showed that hypertension is associated with diminished angiotensinase C gene expression [38]. Angiotensinase C gene mutations with loss of function induced the inadequate degradation of angiotensin II, the final mediator of the RAS, followed by blood pressure elevation [24]. The present findings revealed downregulated angiotensinase C mRNA and protein expressions in the LV tissue of SHRs, a widely studied animal model of human essential hypertension, especially in SHRs with chronic ethanol consumption.

## 5. Conclusions

The conscious instrumented SHR model represents a clinically relevant, chronic ethanol consumption model for studies of ethanol-induced LV systolic function impairment. With the use of this model, the present study revealed that chronic ethanol ingestion produced progressive LV systolic functional impairment, a downward trend in the production of collagen in LV myocardium, and upregulated RAS activation, which was paralleled by downregulated expressions of angiotensinase C mRNA and protein. This indicates a key role for angiotensinase C acting through the enhanced activation of an RAS-induced low production of LV collagen during the development of LV dilation and systolic dysfunction after chronic ethanol consumption.
